# Introducing diabetes emotional well-being into routine consultations for adults with type 1 diabetes

**DOI:** 10.1007/s44357-026-00010-w

**Published:** 2026-08-31

**Authors:** Ruth Harris, Sarah Sims, Mette Due-Christensen, Vibeke Stenov, Ruth Abrams, Clara Fabian-Therond, Shalini Ahuja, Siobhan O’Connor, Iliatha Papachristou Nadal, Megan Peck, Jane Speight, Richard I. G. Holt, Jackie Sturt, D-Stress Study Group, Jennifer Mohammadi, Joerg Huber, Gary Hickey, Kate Hardenberg, Francesca Fiorentino, Lawrence Fisher, Jennifer Halliday, Ramzi Ajjan

**Affiliations:** 1 Florence Nightingale Faculty of Nursing, Midwifery and Palliative Care, King’s College London, London, UK; 2 Steno Diabetes Center Copenhagen, Copenhagen, Denmark; 3 NAMU - National Center for Applied Research in Older Citizens Living With Multimorbidity, University College Absalon, Roskilde, Denmark; 4 School of Health Sciences, University of Surrey, Guildford, UK; 5 Institute for Health Transformation, School of Psychology, Deakin University, Geelong, VIC Australia; 6 The Australian Centre for Behavioural Research in Diabetes, Diabetes Victoria, Melbourne, VIC Australia; 7 Human Development and Health, Faculty of Medicine, University of Southampton, Southampton, UK

**Keywords:** Diabetes distress, Realist process evaluation, Realist review, Routine consultation, Type 1 diabetes

## Abstract

**Aims/hypothesis:**

Diabetes distress affects approximately 40% of adults with type 1 diabetes and is associated with impaired self-care, but is rarely assessed or addressed in routine care. The D-Stress study aims to develop an innovative care pathway to manage diabetes distress. A core component seeks to integrate comprehensive distress assessment with discussion into routine consultations. This rapid realist review examined how, why and in what contexts diabetes distress assessment and discussion might work to assess, manage and reduce diabetes distress among adults with type 1 diabetes.

**Methods:**

Following guidance for rapid realist reviews, we synthesised evidence from 35 documents identified via expert-led searches and stakeholder consultation. Data were analysed to develop programme theories explaining how the intervention might operate in clinical practice.

**Results:**

Eight programme theories were generated. Key mechanisms include: (1) embedding emotional health in routine care; (2) comprehensive assessment and interpretation for timely support; (3) facilitating self-reflection and priority setting; (4) maintaining continuity through regular emotional health discussions; (5) supporting healthcare professionals to personalise consultations; (6) clarifying care pathways and expectations; (7) normalising emotional responses to type 1 diabetes; and (8) promoting person-centred, collaborative consultations. Evidence indicated that validated type 1 diabetes assessments with structured dialogue can normalise emotional responses and promote person-centred care. Critical contextual factors include sufficient consultation time, staff training and clear referral pathways.

**Conclusions/interpretation:**

Comprehensive assessment with discussion may address a gap in diabetes care by integrating emotional health into routine consultations. The programme theories developed here suggest that diabetes distress assessment and discussion may reduce risk of escalation of distress, empower adults with type 1 diabetes and strengthen therapeutic relationships. These findings will guide the realist process evaluation of the D-Stress pathway, highlighting contextual considerations essential for implementation.

**Graphical abstract:**

**Figure:**
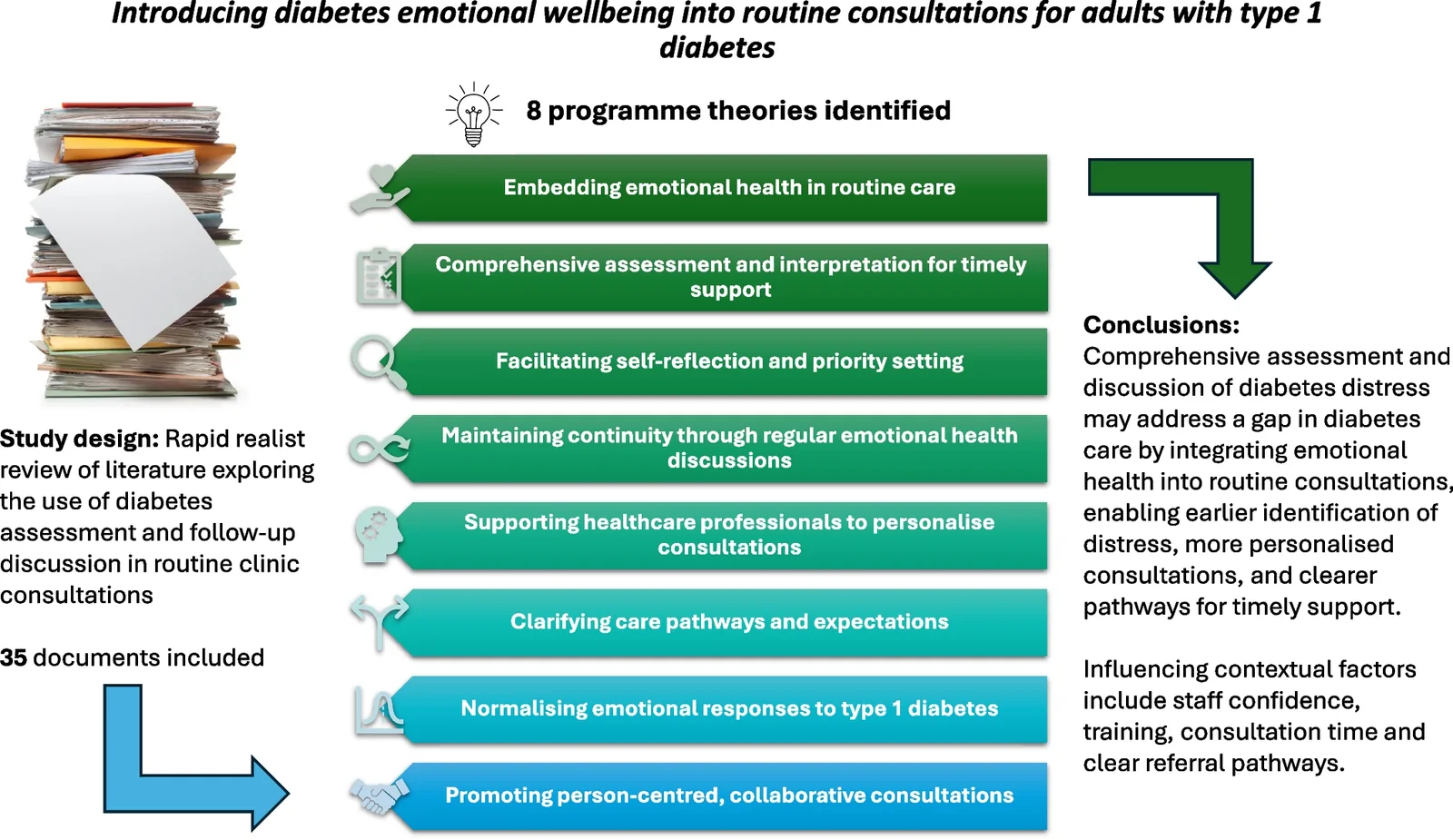

**Supplementary Information:**

The online version contains supplementary material available at https://doi.org/10.1007/s44357-026-00010-w.

**Figure:**
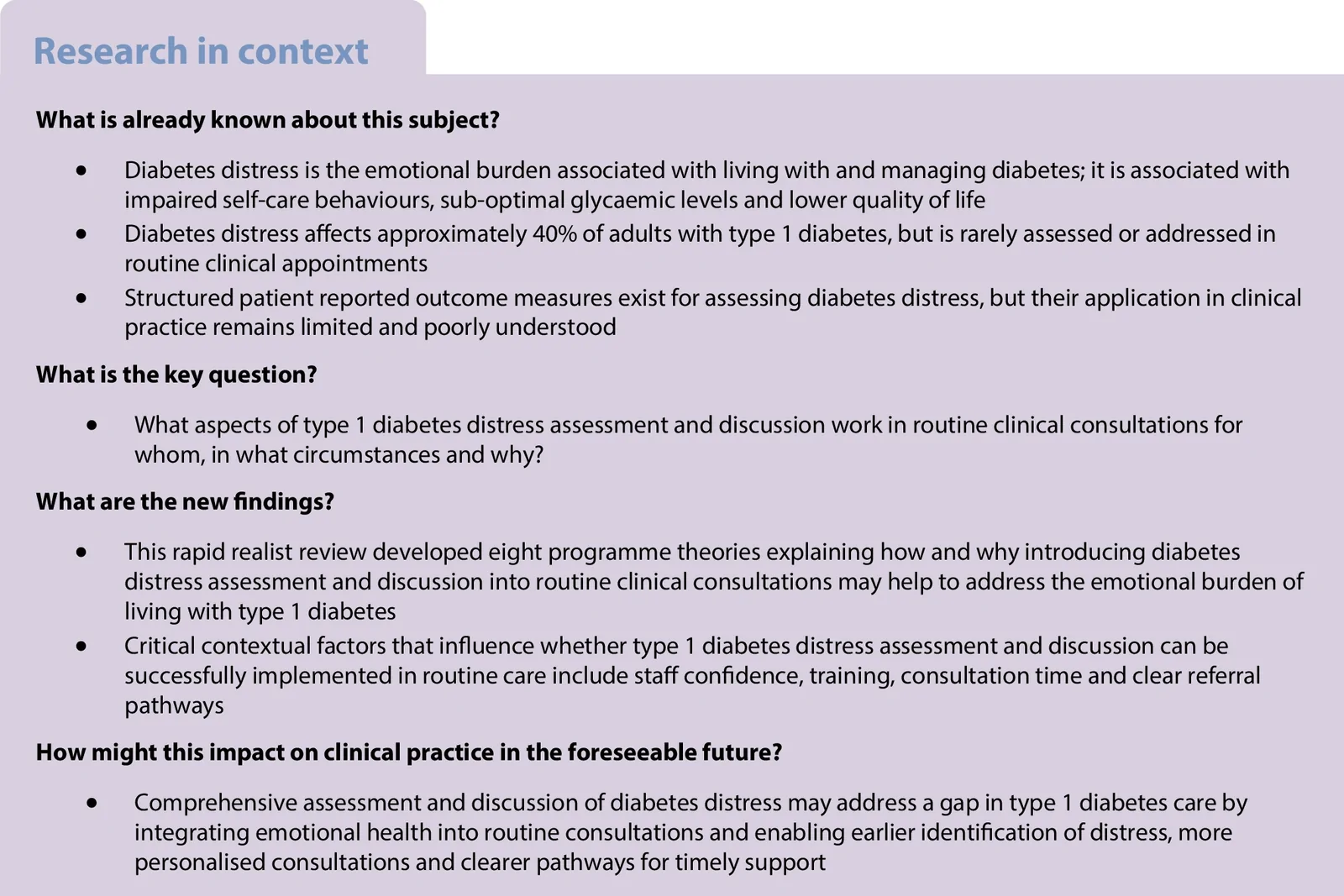

## Introduction

Diabetes distress is the emotional burden associated with living with and managing diabetes, including worries, feelings of overwhelm, frustration, isolation and perceived inadequacy in managing diabetes [1]. It is associated with impaired self-care behaviours, sub-optimal glycaemic levels and lower quality of life [2, 3]. Without appropriate assessment and support, diabetes distress can contribute to acute complications (e.g. hypoglycaemia and diabetic ketoacidosis) and long-term complications (e.g. nephropathy) [4, 5]. Diabetes distress is distinct from depression or general psychological distress and is highly prevalent, affecting around 40% of adults with type 1 diabetes [6]. Recent evidence suggests that diabetes distress is even more widespread, with up to 80% of adults with diabetes reporting at least one problem area that could contribute to diabetes distress (e.g. management difficulties, worrying about complications, hypoglycaemia, financial worries and technology challenges) [7]. Despite this high prevalence, diabetes distress is rarely assessed and managed in routine diabetes care [7, 8]. Prolonged distress can lead to diabetes burnout — a state of emotional and physical exhaustion that reduces engagement with diabetes care and self-management [5] and is associated with increased healthcare costs [9, 10]. Reducing diabetes distress improves diabetes self-management, enhances quality of life, increases healthcare engagement and helps to mitigate rising health costs [6, 9].

This significant gap in diabetes care has prompted recent funding calls by the US National Institutes of Health and the UK National Institute for Health and Care Research and Diabetes UK to commission research into diabetes distress. One of these commissioned research projects, the D-Stress study (https://fundingawards.nihr.ac.uk/award/NIHR205441), is developing and integrating an innovative care pathway to manage diabetes distress in adults with type 1 diabetes in the UK National Health Service. Although diabetes distress is also widespread in adults with type 2 diabetes, this 5-year programme of study focuses only on adults with type 1 diabetes, as the contextual factors surrounding the sources and drivers of distress and the prevalence and stability of distress are recognised to be different [6]. The differences in management strategies between the two types of diabetes are also likely to impact the experience of diabetes distress [11]. Furthermore, type 2 diabetes-specific distress is often related to lifestyle changes and stigma surrounding preventability, complex medical intensification (e.g. transitioning to insulin) and health inequalities (e.g. lack of access to diabetes technology) [12, 13].

One component of the D-Stress care pathway, which is called ‘Enhanced Usual Care’ in the D-Stress study, seeks to address this unmet need by detecting, assessing and discussing diabetes distress within routine diabetes clinic appointments. The aim is to reduce the risk of escalation of distress over time among those with sub-threshold levels of diabetes distress on the assessment tool being used and to refer those with higher distress to targeted support. Clinical diabetes guidelines emphasise the critical need to integrate assessment of diabetes distress into care using a validated patient-reported outcome measure (PROM) of diabetes distress [14]. Several diabetes distress assessment tools exist. These were developed and validated for the twin purposes of enhancing clinical care and as a research tool. Their use in research, to evaluate interventions and determine the prevalence of diabetes distress, has been far more widespread than in clinical care. Limited evidence exists for their use as a clinical tool to support emotional-focused discussions on living with diabetes. The T1‑Diabetes Distress Assessment System (T1‑DDAS) [15] was selected for use in the D-Stress study because it is the most recent, validated and type‑1‑specific measure, published in 2024, and designed explicitly to capture both overall intensity and distinct sources of diabetes distress in adults with type 1 diabetes. It includes an 8‑item CORE scale capturing the overall intensity of diabetes‑related distress, along with 10 SOURCE scales assessing specific contributors to distress.

The aim of this rapid realist review was to develop programme theories explaining how and why introducing comprehensive assessment and discussion of diabetes distress into routine consultations may help to address the emotional burden of living with type 1 diabetes, reduce risk of escalation and identify when additional support is needed.

This type 1 diabetes rapid realist review was conducted as part of the funded D‑Stress programme to inform the timely co-adaptation of the intervention, with its theories intended for testing in a subsequent realist process evaluation. A realist review of diabetes distress assessment and discussion for people living with type 2 diabetes is published in a separate paper in *Metabologia* [16].

## Methods

**Fig. 1:**
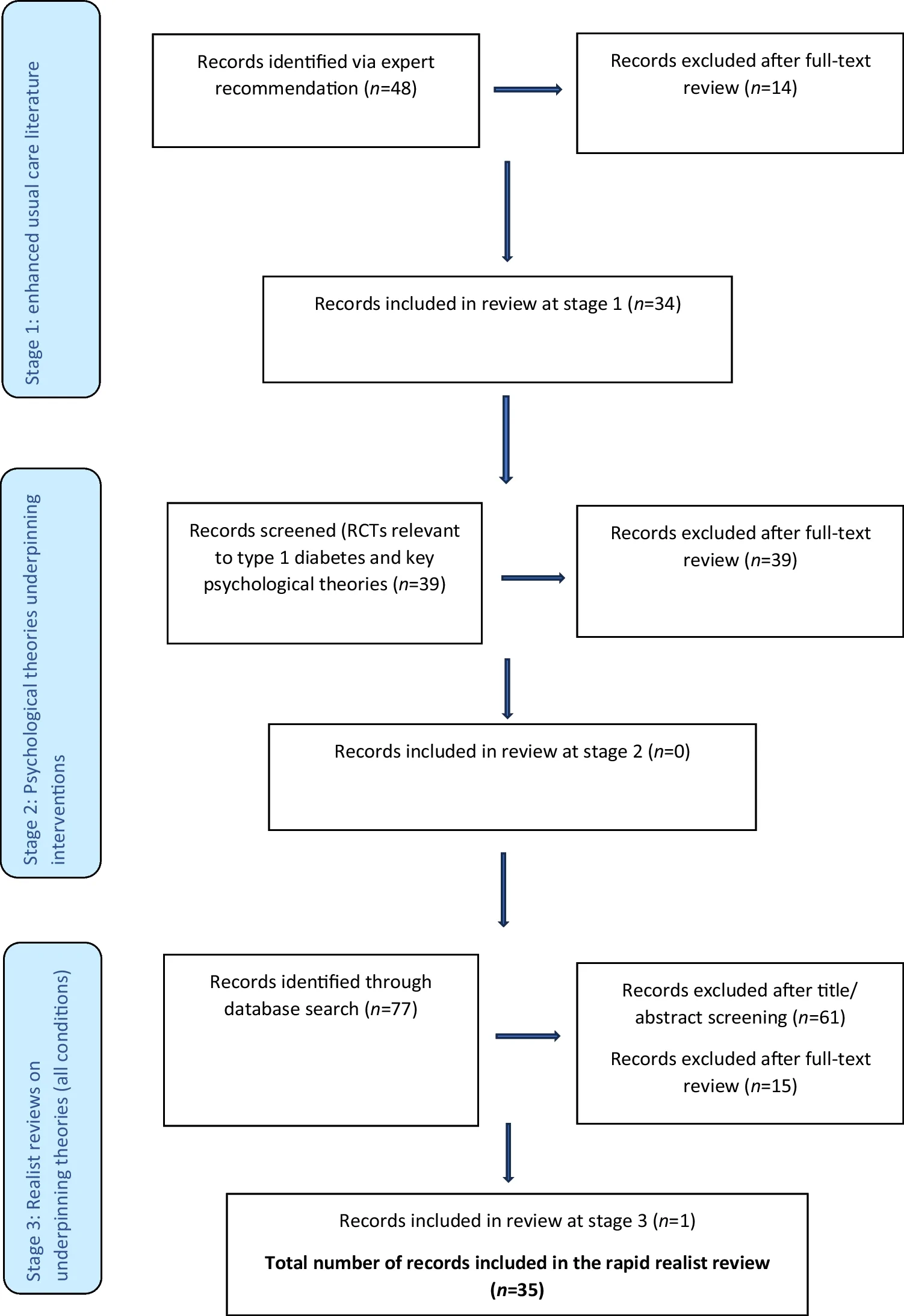
Flowchart of the rapid realist review process

A rapid realist review methodology was selected for several reasons. First, the diabetes distress assessment and discussion intervention is seen as a complex, social intervention that may be delivered differently by different healthcare professionals (HCPs), in different clinic settings and received in varying ways by different people. Second, development of evidence-based context–mechanism–outcome configurations (CMOCs) would contribute a theoretical framework to support a realist process evaluation for a subsequent main trial, which had a fixed time schedule. Evidence-informed guidance for conducting rapid realist reviews was followed [17] and quality and publication standards in realist reviewing were upheld [18, 19]. Definitions of how realist terms were used in this review [20] are provided in Table 1. A flow diagram for the realist review process is provided in Fig. 1.

**Table 1:** Definitions of realist terms and how they were used in this rapid realist review

Realist term	Definition
Programme theory	Specifies what is supposed to be done in an intervention or provision of service (theory of action) and how and why that is expected to work (theory of change) Wong et al. [20]
Context (C)	*The ‘backdrop’ of programmes and research… broadly understood as any condition that triggers and/or modifies the behaviour of a mechanism* Jagosh et al. [21]
Mechanism (M)	*…underlying entities, processes or structures which operate in particular contexts to generate outcomes of interest.* Astbury et al. [22]
Outcome (O)	The result of the interaction between a mechanism and its triggering context Greenhalgh et al. [23]
Retroduction	*Retroduction refers to the identification of hidden causal forces that lie behind identified patterns or changes in those patterns… Retroduction uses both inductive and deductive logic, as well as insights or hunches. It involves thinking through what causal powers might be at work in producing observed patterns or changes in patterns. It is underpinned by a belief that an understanding of causation cannot be achieved using only observable evidence. Retroductive theorising requires that inquirers use their common sense, intelligence, expertise, and informed imagination to build and test theories about underpinning causal processes* Greenhalgh et al. [24]

The scope of the review focused only on literature related to: (1) adults with type 1 diabetes; and (2) specific interventions under investigation in the D-Stress study, alongside the key psychosocial theories underpinning them. Thus, the research question identified for the review was: what aspects of comprehensive assessment of diabetes distress and discussion of emotional well-being in routine clinic consultations work, for whom, in what circumstances and why?

The review was undertaken using a three-stage literature searching process, incorporating stakeholder consultation throughout. Stakeholders included programme architects, adults with type 1 diabetes, diabetes practitioners and methodological experts. As an evidence review of secondary sources we did not consider sex and gender in the review design.

### Stage 1: literature relating directly to diabetes distress assessment and discussion interventions

An exhaustive search was not undertaken in order to streamline and accelerate the search process, as per rapid realist review guidelines [17]. Instead, all study investigators, the majority of whom have expertise in diabetes distress, were asked to identify relevant articles and documents that they thought should be included in the review. Inclusion criteria were that papers must discuss type 1 diabetes, be relevant to assessing or addressing diabetes distress in routine management or highlight evidence of any associated context, outcome or mechanism. Any type of published or unpublished document was potentially applicable, including empirical research studies, literature reviews and grey literature, such as best practice guidance, presentations and discussion pieces.

Conventional quality appraisal methods were not applied, but instead, each study was assessed for its ‘fitness for purpose’ by examining its relevance and rigour [25]. In keeping with realist approaches, the focus was not on study design, but on whether the study offered credible, trustworthy insights that contributed to theory development [20, 26]. The lead reviewer (RH) read all papers and other members of the team divided the papers between them as second reviewers, so that every paper was read and discussed by two researchers. A bespoke data extraction sheet was created, and this was completed by every researcher for each paper read. Salient details about each paper were recorded on the data extraction sheet to identify elements that could help to answer the research question. For excluded papers, reasons for exclusion were also recorded. Researchers met to discuss inclusion and exclusion to ensure that agreement was reached.

The researchers independently compiled a list of initial programme theories based on the papers they had read to produce eight overarching programme theories and their associated CMOCs. CMOCs can be described as specific and individual causal explanations of how an intervention produces outcomes in practice (Table 1). On the advice of a member of the review advisory group, these CMOCs were re-worded as ‘if–then-because’ statements, to allow for testable theory building. An if–then-because statement is a structured way of expressing a proposed causal explanation in realist research, which defines the ‘if’ as the relevant context or condition, the ‘then’ as the expected outcome and the ‘because’ as the underlying mechanism that explains why the outcome is likely to occur in that context. These configurations were rarely made explicit in the papers, limiting their applicability. Furthermore, most papers did not examine a specific intervention. Therefore, retroductive analysis and reasoning were used to interpret how outcomes may have been generated by mechanisms in particular contexts. Nineteen ‘if–then-because’ statements were identified from the eight theories (Table 2).

**Table 2:** Preliminary CMOCs for the D-Stress diabetes distress assessment and discussion intervention

CMOC number	CMOC description
1	If the use of comprehensive diabetes distress assessment and conversation within a clinic indicates that HCPs are expected to review diabetes distress self-assessment scores and ask about emotional health, then the HCP will review the assessment score and ask every person with type 1 diabetes about their emotional health during their consultation time because it raises awareness that assessing and managing emotional issues is an expected part of the HCP role in diabetes care
2	If a validated self-assessment tool is used to assess diabetes distress prior to the consultation, then the HCP can ask the person with type 1 diabetes questions about emotional aspects of diabetes and explore reasons for their distress, because they are aware of the person with type 1 diabetes’ level of diabetes distress and the scores for specific sources of distress
3	If the person with type 1 diabetes undertakes the diabetes distress assessment before their clinic appointment, then they are more prepared to talk about their feelings, emotions and concerns about having diabetes in the consultation, which focuses the discussion on actual patient need because the person with type 1 diabetes has had the opportunity to think about how they are feeling before their appointment and is expecting to discuss this with the HCP during the appointment
4	If the person with type 1 diabetes is not aware that diabetes distress is a normal response to having type 1 diabetes and they complete the diabetes distress assessment then they are more likely to come to their consultation prepared to talk how their feelings about diabetes are influencing aspects of their life and how they manage their condition because diabetes distress assessment prompts them to consider and helps them make sense of how they are feeling about living with type 1 diabetes
5	If the clinic appointment is restricted to a short time and discussion of physical health checks is considered more important or the clinic is running late, then the HCP may not ask the person with type 1 diabetes questions about their emotional health because they may think that these discussions are not a priority or that there is not enough time, as they think these conversations can take longer
6	If regular, comprehensive assessment is carried out for all people with type 1 diabetes using a validated self-assessment tool, then low, moderate and high levels of diabetes distress will be identified and addressed either through support from the diabetes team or through referral for additional support, because use of a validated tool for assessment of diabetes distress will give both the person with type 1 diabetes and the HCPs information about the person with type 1 diabetes’ level and source of distress so that they can take action to address it
7	If a HCP receives education in how to use, interpret and discuss a diabetes distress score with a person with type 1 diabetes, as well as training in processes for subsequent action, then accurate assessment of diabetes distress and suitable actions based on this score will be implemented and the person with type 1 diabetes’ informed preferences will be considered, because the HCP will be knowledgeable about diabetes distress and confident to support person with type 1 diabetes to talk about diabetes distress and participate in the consultation
8	If a diabetes service has clear referral pathways and timely availability of specialist services, then HCPs will feel more confident about having conversations about diabetes distress, because they trust that appropriate help is available and feel confident that they can refer individuals who show high levels of diabetes distress to suitable specialist services
9	If a person with type 1 diabetes finds it difficult to reflect on their emotional health and raise these feelings in a consultation, then diabetes distress assessment can help them to prepare for the consultation, decide what they want to discuss in the consultation and feel empowered to talk about what is important to them, because the comprehensive focus on diabetes distress encourages the person with type 1 diabetes to recognise how they are feeling and understand how this may affect their day-to-day life
10	If clinic appointments are time restricted and a short version of a diabetes distress assessment can be used, then it is more likely that a person with type 1 diabetes will have a diabetes distress assessment, although this may not be as accurate as a long version of the assessment, because there can be variation between individual subscales of diabetes distress
11	If emotional well-being is discussed and followed up at each appointment, then the person with type 1 diabetes is more likely to feel comfortable talking about diabetes distress, because talking about diabetes distress becomes normalised as part of a diabetes consultation
12	If the HCP has received training, and the clinic record keeping enables availability of accurate content of previous consultations and diabetes distress assessment scores, then the HCP can be open, kind and positive and support the person with type 1 diabetes to feel comfortable talking about emotional and psychosocial issues, because the HCP feels prepared and confident to talk about the issues raised by the person with type 1 diabetes and is aware of their previous diabetes distress assessments
13	If the person with type 1 diabetes sees a different member of the diabetes team at each appointment, then diabetes distress assessment and discussion ensures that a conversation about diabetes distress is part of the appointment, irrespective of who they see, and ongoing issues are followed up because the regularity of the discussion at each appointment, structured by diabetes distress assessment, promotes continuity of care
14	If HCP members of the diabetes team do not have a psychology background then diabetes distress assessment and discussion intervention supports them to facilitate and tailor the conversation about emotional issues and well-being to better meet the needs of the person with type 1 diabetes and conveys that the HCP cares about how the person with type 1 diabetes is feeling; the diabetes distress assessment and discussion supports the HCP to feel more prepared and confident to have the conversation, because diabetes distress assessment provides a structured guide for HCPs to start an open conversation
15	If a person with type 1 diabetes is unclear about what to expect in their diabetes care, then comprehensive diabetes distress assessment and conversation can help them to manage the emotional and social challenges and be reassured that there is a plan for their care, because diabetes distress assessment and discussion provides a clear pathway of what to expect, which helps them to anticipate the emotional and social responses to life with the condition
16	If a person with type 1 diabetes has feelings of shame, stigma and powerlessness (i.e. feeling unable to cope) associated with diabetes and diabetes distress assessment and discussion routinely flags up the importance of emotional health for people with type 1 diabetes and acknowledges that a diagnosis of type 1 diabetes has a significant psychological impact that makes it more difficult to self-manage, then the person with type 1 diabetes feels more able and comfortable discussing their emotional health during a consultation, because their feelings are normalised as part of their experience and the person with type 1 diabetes feels understood, listened to and ‘seen.’
17	If HCPs use the comprehensive assessment of diabetes distress to guide the conversation using more open questions then the person with type 1 diabetes will feel able to direct the content of the discussion to issues that are important to them, because the person with type 1 diabetes feels empowered to direct the conversation on a more equal, person-centred basis
18	If the HCP feels appropriately skilled to actively listen, then the consultation becomes more person-centred with increased satisfaction for both the person with type 1 diabetes and the HCP, because the person with type 1 diabetes feels more autonomy and responsibility in the discussion to talk about what matters to them
19	If the HCP is worried about not having enough time to spend with the person with type 1 diabetes because consultation time is short or the clinic is running behind time, then they may not enable the person with type 1 diabetes to say everything they want to talk about, because the HCP is distracted, may not be actively listening or may be reluctant to have conversations that may take longer that the time available or that may be left incomplete or unresolved

The team met regularly throughout this process to explore the theories and statements, and all queries were discussed until agreement was reached. Engaging key stakeholders during the process helped us to validate the findings and identify any gaps in the literature. Realist approaches recommend this step, as understanding stakeholders’ insights about the intervention and its intended purpose is vital for explaining how it might work [27]. To operationalise this, we presented stakeholders with the emerging theories and their supporting statements and asked them to reflect on how well they resonated with their own experiences of delivering, receiving or designing the intervention (i.e. whether they felt plausible and whether any mechanisms or contextual factors were missing). Stakeholders were also invited to comment on the clarity of the theory descriptions and identify any requiring refinement. The overall feedback from stakeholders was that the eight theories and 19 if–then-because statements accurately captured the generative mechanisms of the intervention. Some small changes were made to clarify the theory descriptions, but no new theories were identified.

### Stage 2: literature relating to the psychological and social theories underpinning diabetes distress assessment and discussion

We aimed to identify whether there were any randomised controlled trials (RCTs) of diabetes distress-related interventions for people with type 1 diabetes based on the same psychological and social theories underpinning diabetes distress assessment and discussion, self-determination theory and the biopsychosocial model.

We accessed a Covidence database developed for a systematic review [28] underpinning the EASD Diabetes Distress Clinical Guidelines to identify any similar type 1 diabetes distress interventions also underpinned by these psychological and social theories.

### Stage 3: realist reviews exploring interventions outside of diabetes distress based on self-determination theory and the biopsychosocial model

A third and final stage of searching was undertaken to identify any realist reviews or realist evaluations exploring interventions outside of diabetes distress based on self-determination theory and the biopsychosocial model. This approach of looking outside of the scope of the intervention under study is well supported in realist approaches and helps to avoid narrow interpretations [27, 29]. Expert advice was sought from library and information sciences specialists around generating relevant search terms and four electronic databases were searched on 24 February 2025 using the strategies highlighted in Electronic Supplementary Material (ESM) Box 1. This search also included terms to identify psychological theories underpinning interventions relevant to other aspects of the D-Stress study.

Salient details about each included paper identified in stages 2 and 3 were recorded on the data extraction sheet and initial theories and statements in these papers were explored as in stage 1. Although some supporting evidence for the initial theories identified in stage 1 were found in stage 3, no new theories or statements were identified. All the included papers were re-read to explore whether any specific if–then-because statements could be identified within each theory. These statements were again circulated among the study stakeholders and modifications were made, where required. These statements helped to articulate tentative casual pathways to be refined in the subsequent realist process evaluation.

## Results

A total of 35 papers were included across all three search stages. At stage 1, 48 papers were identified: 34 were included [1, 4, 7, 30–60] and 14 were excluded for not being relevant [8, 61–73]. In stage 2, 39 papers were screened but none provided relevant evidence. In stage 3, 77 papers were identified and 61 were excluded at title and abstract screening for not being a realist review, evaluation or synthesis; 16 full-text papers were assessed, one of which was included [74] and 15 of which were excluded for not including comparable interventions to diabetes distress assessment and discussion.

A brief description of each paper is provided in ESM Table 1, and these comprised: published research articles (*n* = 23); an unpublished working research article (*n* = 1); assessment scales (*n* = 5); PowerPoint presentations (*n* = 2); reviews (*n* = 2); and discussion pieces or guidelines (*n* = 2). The papers referred to work undertaken primarily in Denmark (*n* = 10), followed by the UK (*n* = 9), the USA (*n* = 7) and Australia (*n* = 5). The papers were published between 2009 and 2026. The eight programme theories are presented below and the 19 if–then-because statements summarised in Table 2. These provide insights into how comprehensive assessment and discussion of diabetes distress into routine care appointments might be expected to work and why.

### Programme theory 1: embedding emotional health in routine care

Seventeen papers addressed the programme theory that diabetes distress assessment and discussion raises the expectation that routine diabetes care will address emotional health [7, 30, 38, 39, 41–46, 51, 52, 54, 55, 58, 60, 74]. Many diabetes clinics are very busy, and time constraints of consultations means that there is a tendency for issues about blood glucose management and physical checks (e.g. blood pressure, foot pulse) to take priority. Furthermore, HCPs often consider diabetes distress to be a mental health condition that should be managed outside regular diabetes care by specialist mental health practitioners.

This programme theory focuses on the idea that the comprehensive assessment and discussion of diabetes distress within routine care demonstrates to staff and the person with type 1 diabetes that addressing emotional health is an important part of usual, holistic care that should occur at every consultation. This expectation gives HCPs the confidence to address these issues and validates the belief that HCPs are often best placed to provide support for diabetes distress. Assessment and discussion of diabetes distress within routine care increases the expectation to talk about emotional responses and mental health and normalises its importance for both people with diabetes and HCPs.

Having sufficient time to discuss feelings and assess distress is important and may result in improved consultation experiences for people with type 1 diabetes. Where healthcare organisations and diabetes services support or require comprehensive assessment and discussion of diabetes distress, this consolidates the expectation that addressing emotional health and well-being is part of routine diabetes care.

**Figure:**
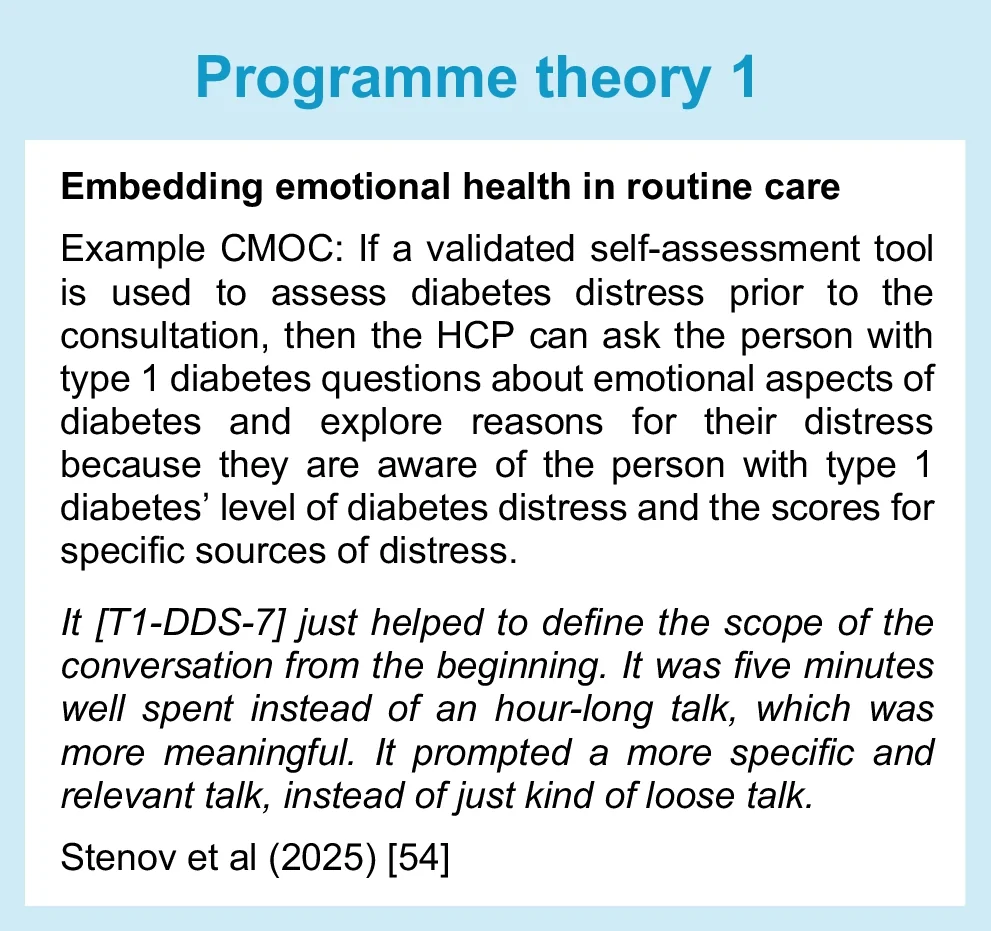

### Programme theory 2: comprehensive assessment and interpretation for timely support

Fourteen papers and five assessment scales addressed this programme theory [7, 30, 31, 38, 39, 42, 43, 46, 48, 50, 52–58, 60, 74]. Regular (often annual) assessment of diabetes distress using a validated self-assessment tool (e.g. T1-DDAS scale) provides a comprehensive assessment of overall diabetes distress to indicate when a person with type 1 diabetes requires support from the diabetes team or specialist emotional and mental health support. This is combined with a clinical review of the total and sub-scale (source) scores to interpret their meaning and how they compare with previous distress scores. This helps the diabetes team to give the right level of support, from discussing management strategies for low levels of distress to further assessment or ongoing follow-up, or specialist referral for moderate-to-high levels of distress. Subscales of diabetes distress scores provide a detailed understanding of how distress is experienced to enable more targeted advice and intervention.

Regular assessment of diabetes distress for all clinic attendees enables consistency to ensure that everyone who has distress is identified, thus enabling timely support to reduce distress and ensure no-one is missed. Having a clear and agreed upon process for each team member’s role in assessing distress supports consistency in capturing all people with type 1 diabetes with high distress and provides a comprehensive and complete referral for specialist help. Adequate recording of diabetes distress scores (e.g. electronic record) ensures that assessment history is captured and that no elevated score is overlooked.

Timely intervention for elevated distress requires having clear referral pathways and available specialist services to which clinicians can refer. Education and training to increase HCP knowledge of the use and interpretation of distress scores, discussion with the person with type 1 diabetes and processes for subsequent action support the implementation of agreed processes. Training may need to be repeated to support new practices to become embedded into and sustained within usual practice.

**Figure:**
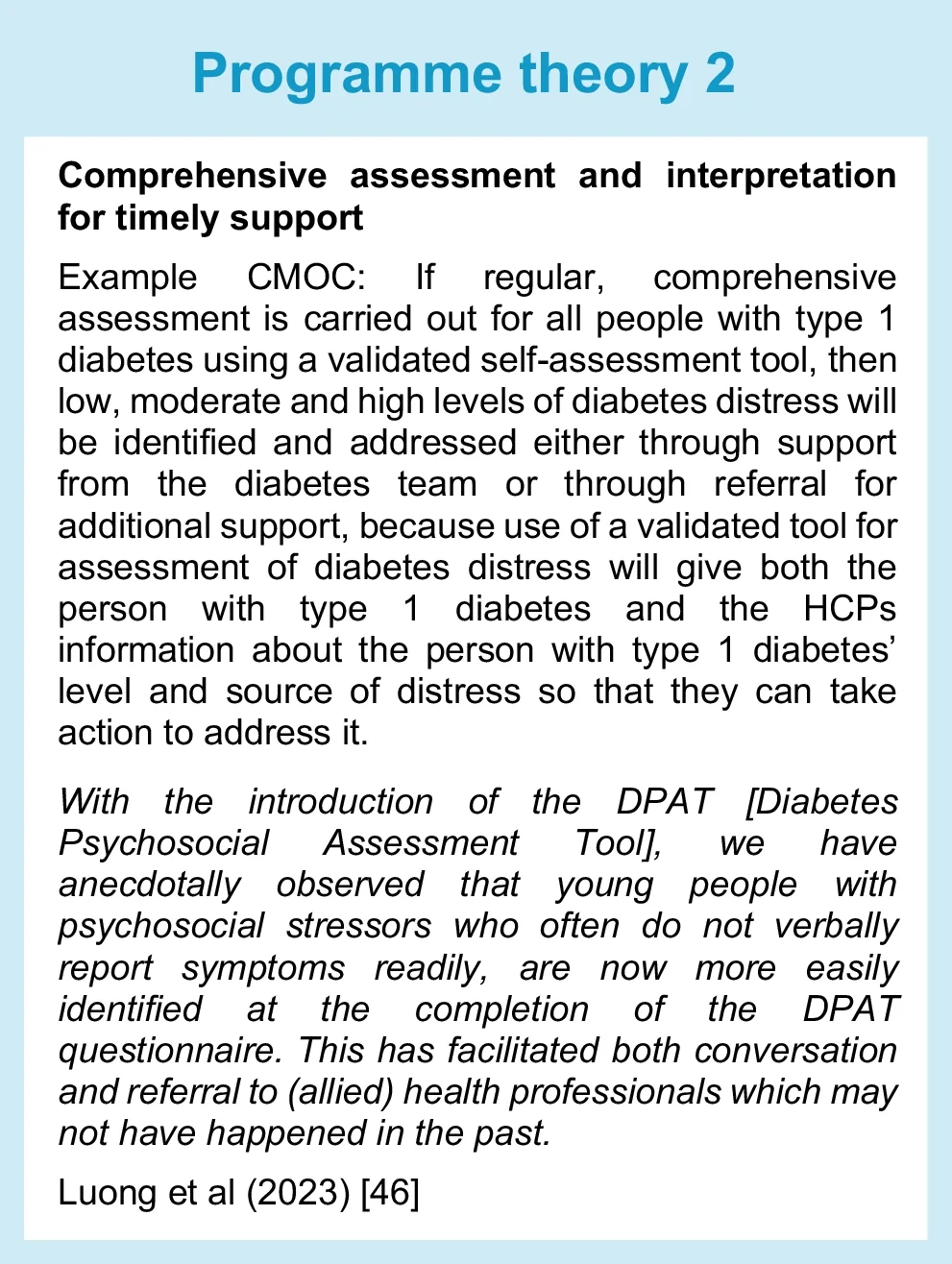

### Programme theory 3: facilitating self-reflection and priority setting

Seventeen papers addressed the programme theory that comprehensive assessment and discussion enables people with type 1 diabetes to recognise how they are feeling and to identify what they want to talk about during their clinic consultation, highlighting the importance of their self-awareness in addressing diabetes distress [1, 4, 30, 34–38, 45, 51–55, 58–60]. Some people with type 1 diabetes find it difficult to reflect on their emotional health and well-being or to raise how they are feeling in a diabetes clinic consultation.

Comprehensive assessment of diabetes distress encourages people with type 1 diabetes to self-reflect and recognise how they are feeling, recognise the range of feelings they are experiencing and acknowledge the impact that diabetes has on their emotional well-being and day-to-day life. By recognising the connection between biopsychosocial aspects of diabetes, the person with type 1 diabetes can become more aware of how thoughts, feelings and behaviours affect self-management and understand their feelings and reactions, which may help them to be better prepared for future experiences and issues. This self-reflection can help people with type 1 diabetes to explore their own values, support them to make decisions related to self-management and improve their communication with HCPs.

Completing a validated diabetes distress assessment tool before a clinic appointment can help people with type 1 diabetes to prepare for the appointment and decide what they want to discuss with the HCP. This preparation may increase patients’ confidence and help them to feel empowered to raise their priorities for discussion, encouraging feelings of self-compassion about when self-management routines do not appear to work and enabling patients to make changes to how they self-manage their condition. This priority setting supports the content of the consultation to be more person-centred. Assessment scores can act as a starting point to open clinical conversation to address often hidden or less talked about micro-dynamics that contribute to diabetes distress, supporting recognition and articulation of feelings of isolation, exclusion, loneliness and shame to enable these to be shared and addressed. Sufficient time in a consultation and the knowledge, skills and confidence of the HCP are important contexts.

**Figure:**
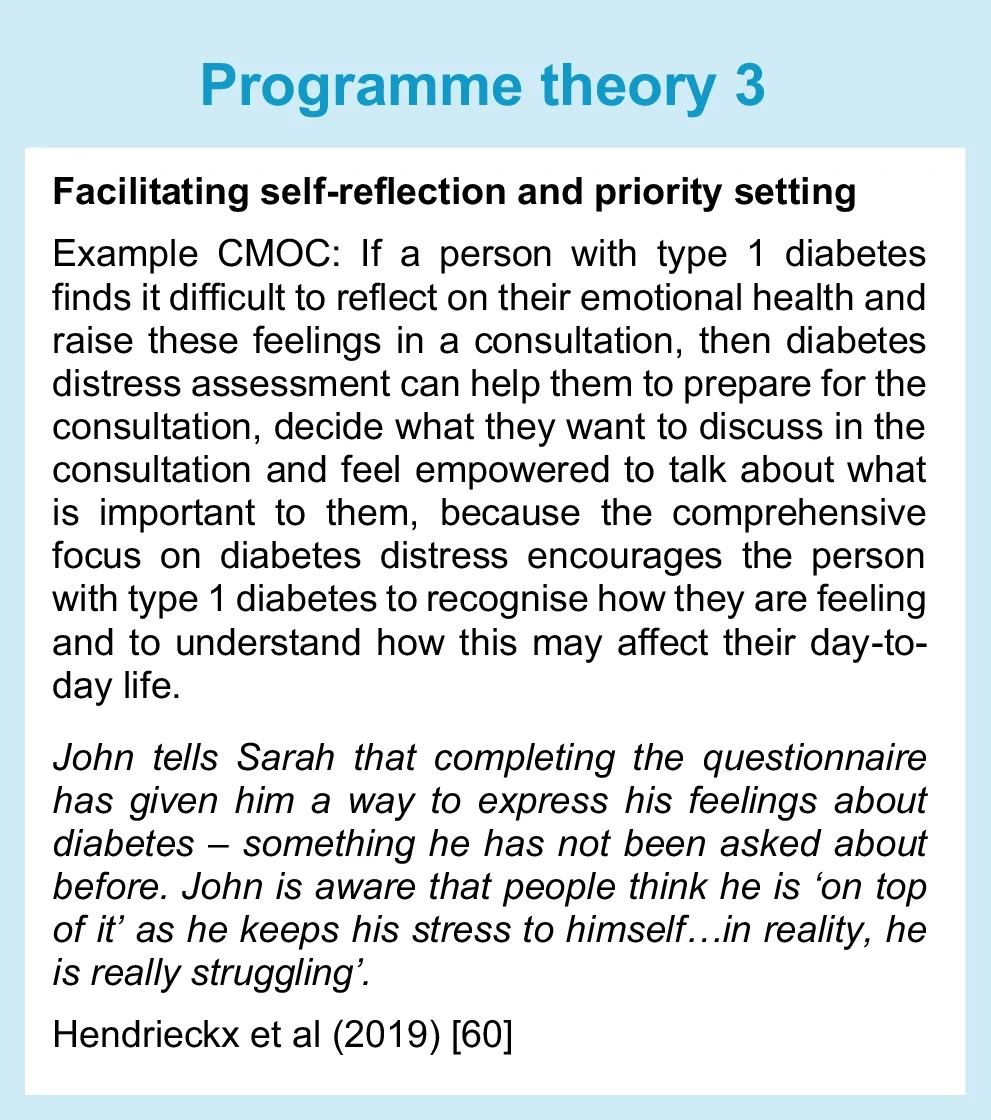

### Programme theory 4: maintaining continuity through regular emotional health discussions

Eleven papers provided evidence of how regular discussion of diabetes distress in routine consultations can enable continuity of care [30, 34, 35, 38, 40, 45, 51, 54, 58, 60, 74]. Regular discussion of the emotional response and experience of having type 1 diabetes legitimises diabetes distress as an important issue that influences how people live with diabetes. This regularity of discussion, structured by an intervention and informed by a structured diabetes distress PROM, supports continuity of care. The person with type 1 diabetes feels that conversations are part of a process irrespective of who they see in the consultation. This also means that they do not have to start from scratch at each appointment and that ongoing issues will be followed up. This continuity enables a safe and trusting interaction and relationship between the HCP and people with type 1 diabetes, which is important, so the person with type 1 diabetes feels comfortable talking about diabetes distress.

HCPs feeling prepared and confident to talk about emotional and psychosocial issues is an important context to enable them to be open, kind and positive. This is helpful for clinic services with a high turnover of staff and resulting low continuity of consulting staff. Good record keeping of the matters discussed in a consultation (e.g. electronic record) is important so that HCPs are aware of what was discussed previously and can follow up on issues, reinforcing continuity of care.

**Figure:**
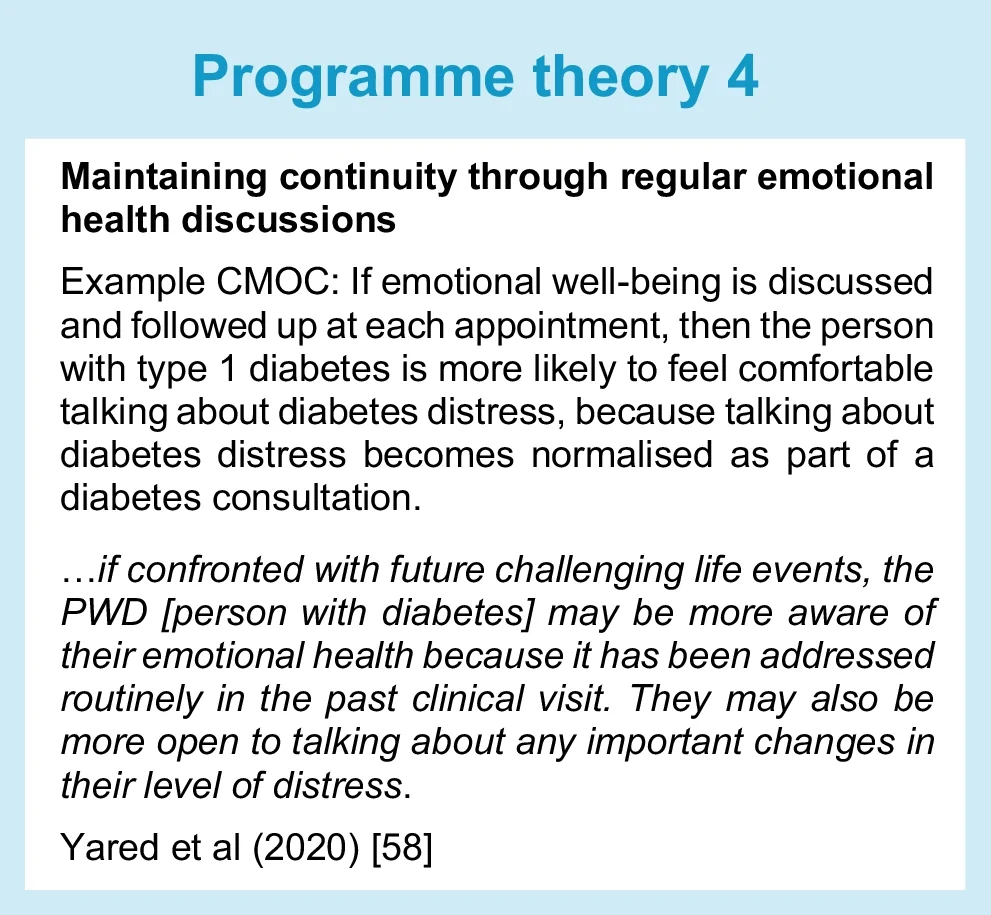

### Programme theory 5: supporting HCPs to personalise consultations

Seventeen papers highlighted how comprehensive diabetes distress assessment and discussion guides can support HCPs to tailor the clinic conversation to the person with type 1 diabetes’ own issues and needs [7, 30, 33, 35, 37, 38, 42, 45–47, 51–54, 58, 60, 74]. Providing a structured guide gives HCPs a conversation starter for an open conversation about emotional well-being during a clinical consultation so that they are able to give the person with type 1 diabetes the opportunity to express and process the biomedical, emotional, psychological and social aspects of diabetes that are relevant to them.

Receiving diabetes distress scores before seeing the person with type 1 diabetes in the clinic can increase the HCP’s understanding of their emotional status and awareness of their priorities to be covered in the appointment. This leads to a consultation that is tailored to the person with type 1 diabetes and that better meets their needs, which can result in increased satisfaction. HCPs conveying the message that they care about how the person is feeling (not only about their physiological test results) strengthens the relationship, rapport and trust between HCPs and the person with type 1 diabetes. This pathway supports HCPs without a psychology background to facilitate a conversation about emotional and psychological issues and adapt to patient needs related to their everyday life, enhancing the therapeutic interaction between the person with type 1 diabetes and the HCP and further supporting a more open dialogue.

**Figure:**
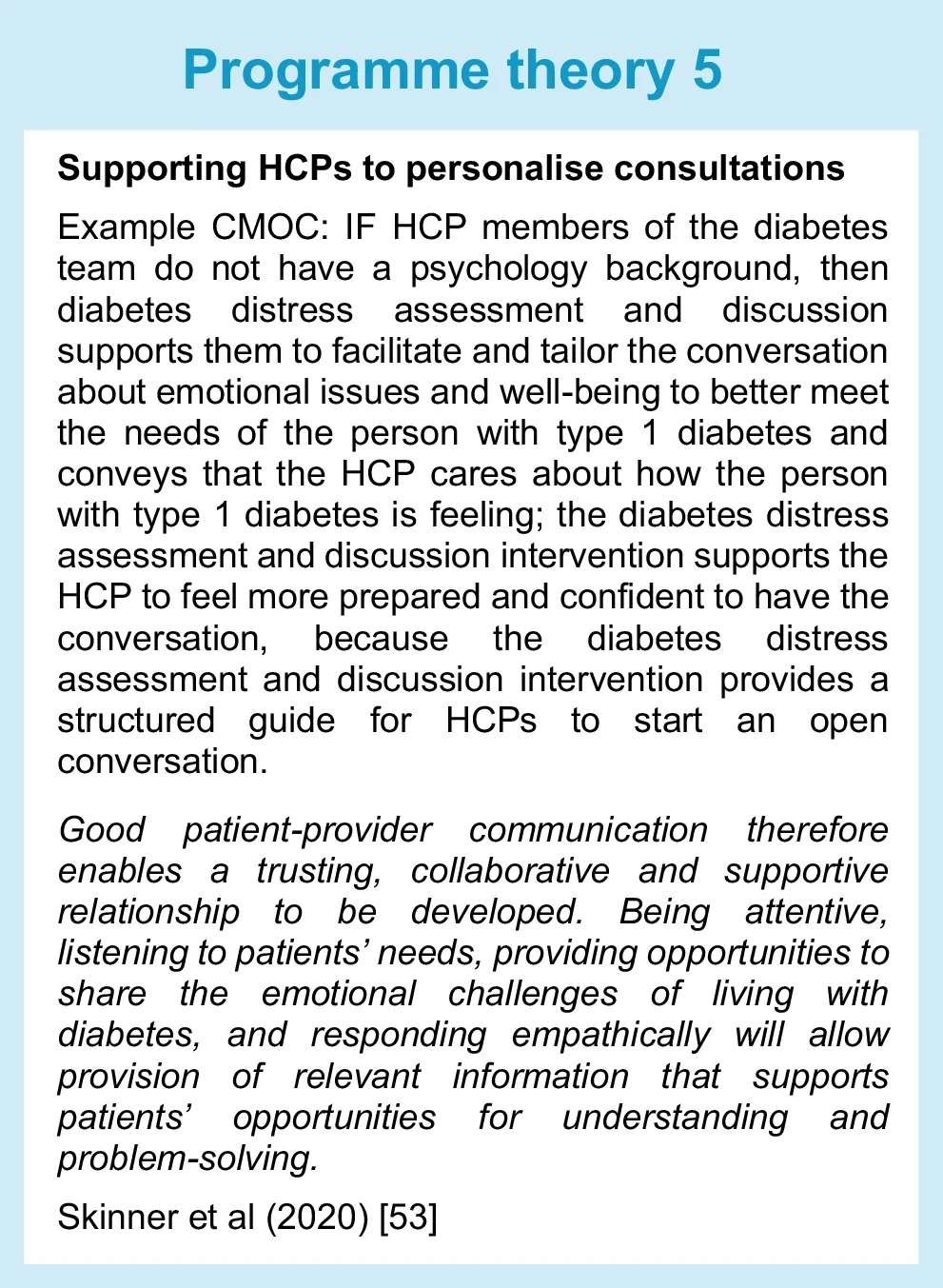

### Programme theory 6: clarifying care pathways and expectations

Five papers addressed how diabetes distress assessment and discussion can signpost and clarify what can be expected from diabetes care [34–37, 60]. These studies provide evidence from the development of consultation tools for use in people with type 1 diabetes during the first year following diagnosis. These tools provide a clear pathway for what a person with type 1 diabetes can expect, helping them to anticipate emotional and social responses to life with diabetes and to be reassured that there is a plan for their care. If HCPs are trained in delivering structured guidance and people with type 1 diabetes receive consistent follow-up, then this guidance and signposting can help them to manage the emotional and social challenges more effectively.

**Figure:**
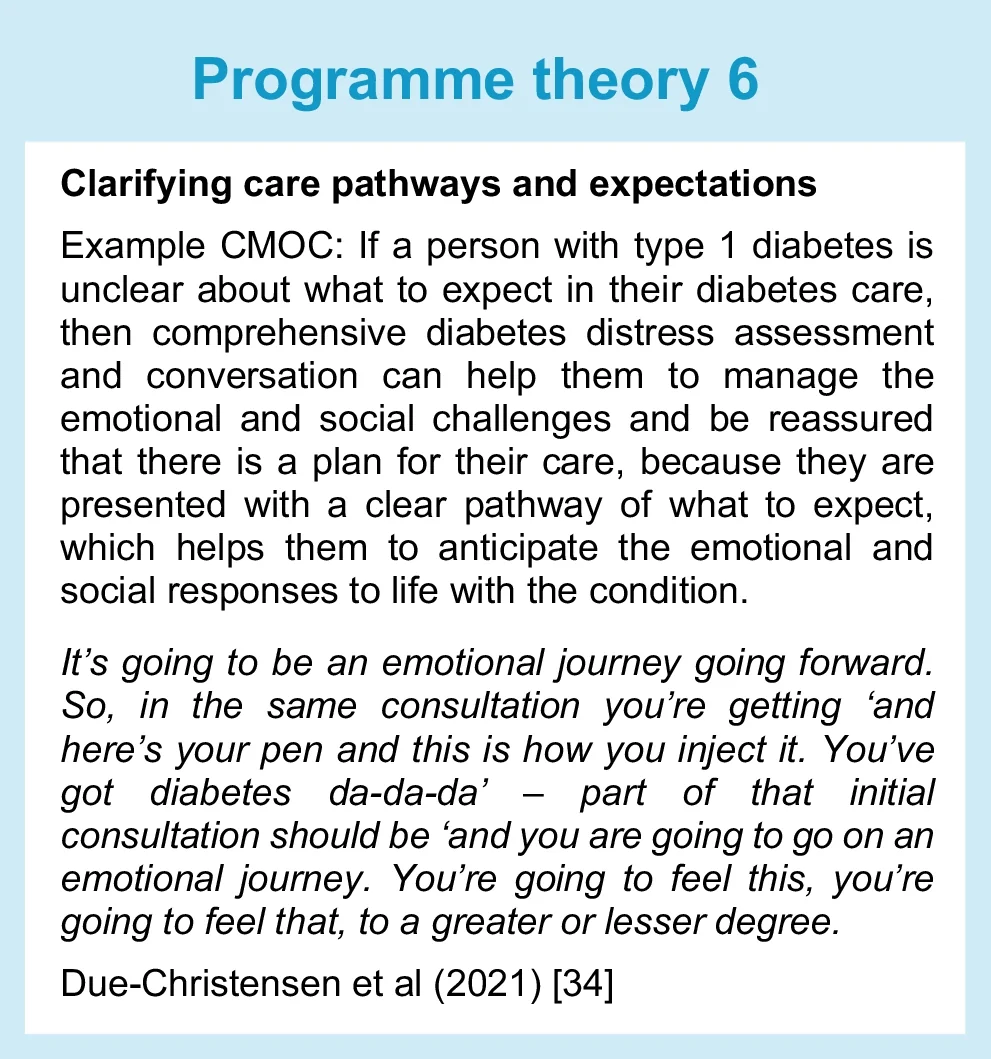

### Programme theory 7: normalising emotional responses to type 1 diabetes

Fifteen papers addressed how the use of a diabetes distress assessment with conversation in the clinic recognises that there is an emotional toll to living with diabetes that is experienced by many [1, 30, 32, 34, 35, 37, 38, 45, 49, 52–55, 58, 60]. This acknowledgement that living with type 1 diabetes has a significant psychological impact, which in turn influences self-management of all aspects of diabetes, helps to legitimise the importance of diabetes distress and emotional support as a routine part of care and follow-up.

Routinely flagging the importance of emotional health in type 1 diabetes care encourages the person with type 1 diabetes to feel that it is normal to feel the way they do and appropriate to seek help when they need to. Normalising thoughts and feelings helps to reframe challenges and focus on solutions to adapting to life with diabetes. HCPs being open to talk about diabetes distress helps to share the burdensome nature of living with and managing diabetes, which is a key facilitator to allow people with type 1 diabetes to feel more supported in discussing concerns and may also ameliorate any guilt, shame and negativity about difficulty in self-management. This also helps HCPs to provide guidance, support and appropriate referral where needed.

**Figure:**
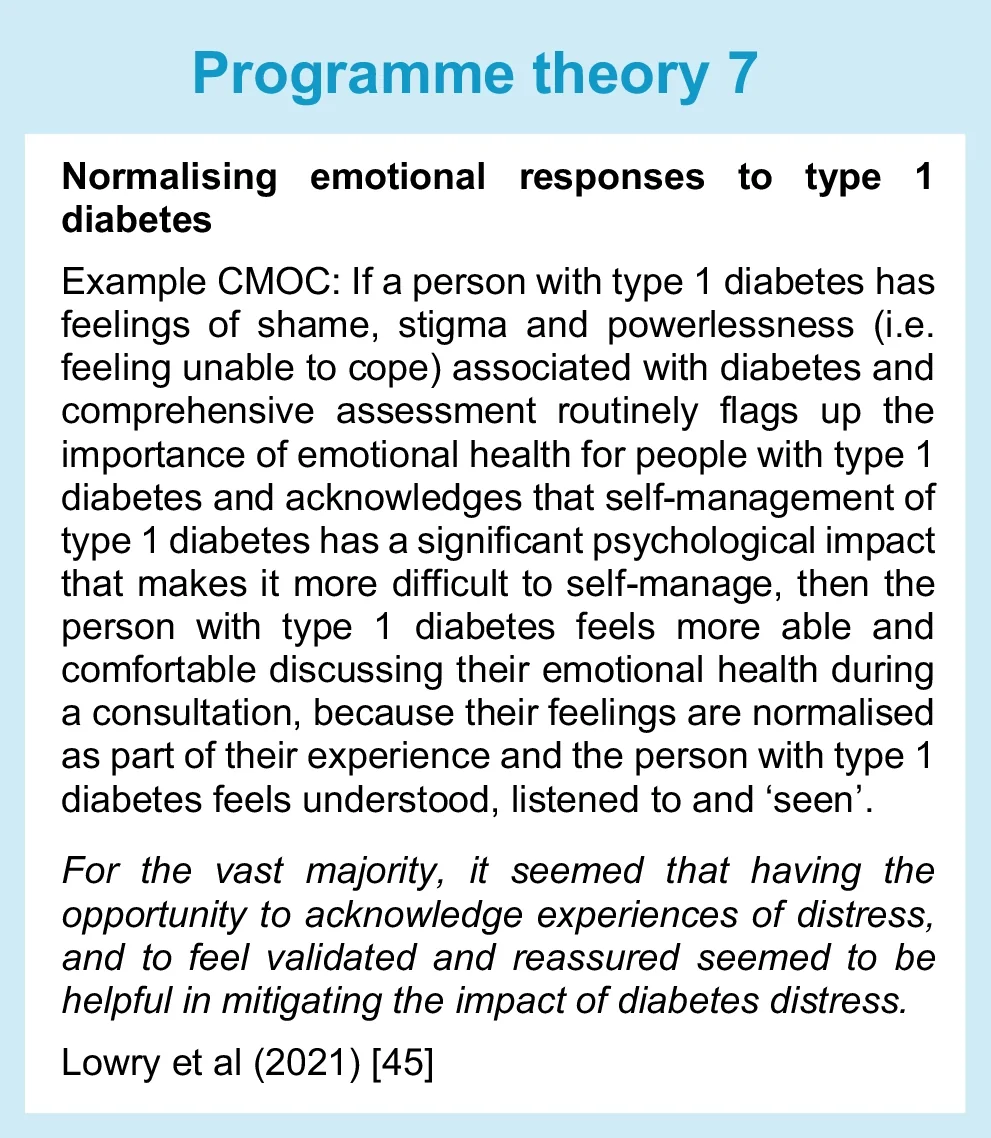

### Programme theory 8: promoting person-centred, collaborative consultations

Nine papers addressed how comprehensive assessment-directed conversation can promote person-centred care [30, 34, 35, 38, 44, 46, 51, 52, 54]. Typically, there is a hierarchical model of interaction between people with type 1 diabetes and HCPs during a clinic consultation, with HCPs tending to spend a large amount of time talking and providing people with type 1 diabetes with information and guidance.

Diabetes distress assessment and discussion require a more balanced conversation, in which HCPs spend less time delivering information and problem solving and more time actively listening and exploring people with type 1 diabetes’ experiences to enable person-centred care. In these interactions, HCPs use open-ended questions and pay more attention to their body language, such as ensuring that they face the person with type 1 diabetes directly and turn away from computer screens. Common concerns are that HCPs may not feel appropriately skilled to undertake such discussions or may worry about the limited time available to spend with people with type 1 diabetes and the fear that addressing such concerns will increase visit time. However, many HCPs find these types of discussion more satisfying in relation to delivering person-centred, personalised care.

**Figure:**
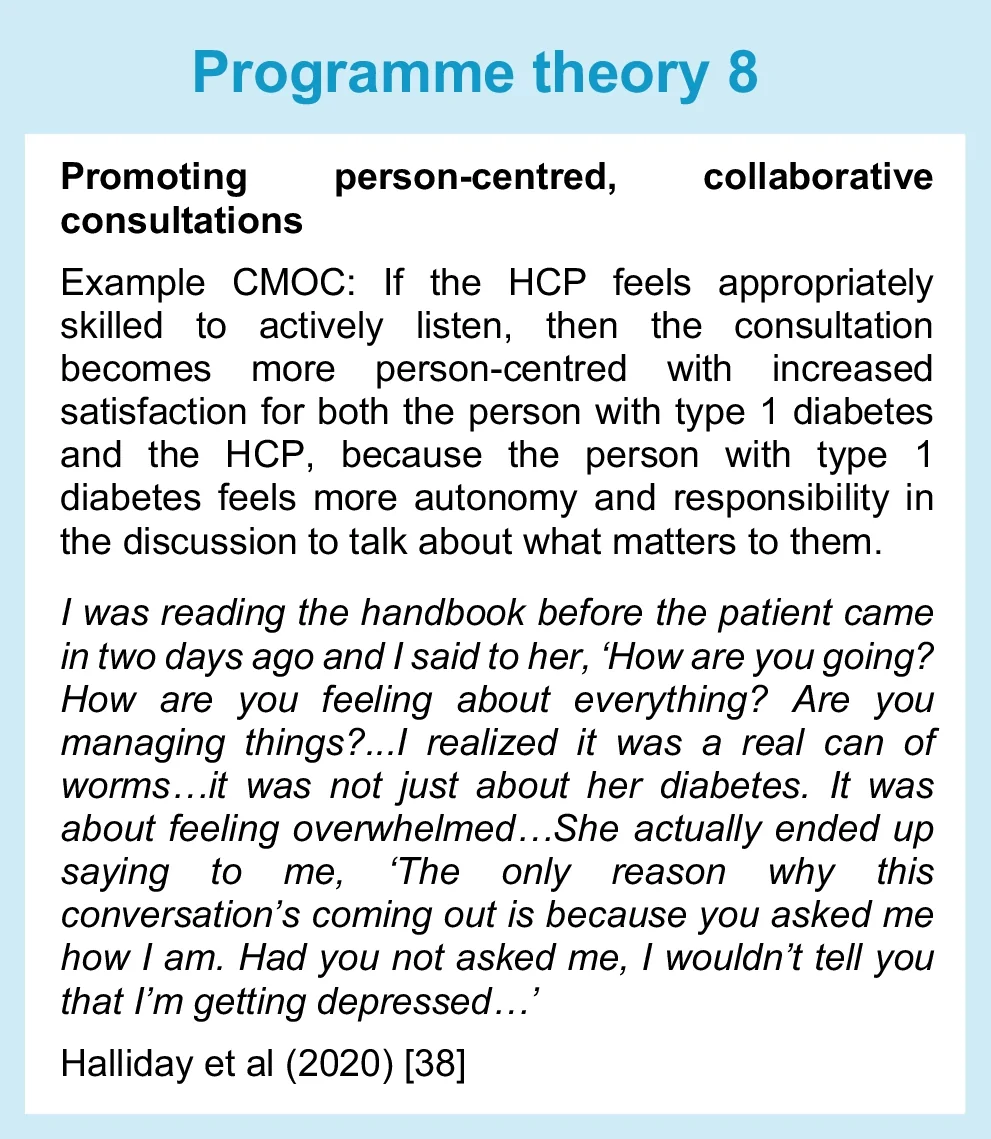

## Discussion

This rapid realist review developed programme theories explaining how and why introducing comprehensive assessment and discussion of diabetes distress into routine consultations may help to address the emotional burden of living with diabetes, reduce risk of escalation and identify when additional support is needed. Evidence about assessment and psychosocial support interventions and their role in supporting emotional well-being in routine consultations was synthesised to identify the mechanisms through which comprehensive assessment and discussion may influence diabetes care outcomes. Although each theory is distinct, there was some overlap and interaction between them [23, 75]. This is common for complex interventions, which rarely work in a linear way.

Several programme theories were supported, although the strength of evidence varied. In line with realist principles, the strength of evidence did not refer to a quantitative rating but the extent to which empirical findings from diverse study types consistently supported, refined or challenged a given programme theory. Evidence was judged as stronger where multiple, methodologically varied studies offered coherent support for the same causal explanation, and weaker where evidence was sparse or insufficiently detailed to illuminate theory. Stronger evidence related to the value of regular, comprehensive distress assessment as a resource for both HCPs and people with diabetes informing person-centred conversations and guiding advice and support. There was also substantial evidence that diabetes distress assessment and discussion can normalise diabetes distress, reassuring people with diabetes that emotional challenges are a legitimate and expected part of routine care. The findings emphasise the importance of sufficient consultation time and of HCPs being skilled and confident in asking about emotional well-being and responding supportively.

Some theories, such as the role of signposting and clarifying expectations of diabetes care were supported by less evidence. However, this does not necessarily indicate that these mechanisms have a reduced impact on addressing diabetes distress but that existing studies have not examined them in sufficient depth. A notable finding of this review was that the stage 2 search, which focused on identifying evidence from RCTs, yielded no eligible papers. This suggests that theory‑driven trials examining emotional well-being assessment in diabetes care are rare, and that mechanisms are often implied rather than explicitly articulated or tested, highlighting the need for research that makes causal processes explicit and that empirically examines how and why distress assessment influences outcomes. This absence strengthened the rationale for adopting a realist approach, as retroductive reasoning was required to develop and refine programme theories in an area in which mechanisms were under‑specified. Future work, including the D‑Stress feasibility study, will be essential in addressing this gap by testing these programme theories in clinical practice and identifying which mechanisms operate, for whom and in what contexts. It is possible that some theories will not be supported in clinical practice or that alternate theories will emerge. This consideration of context and local implementation factors, which may include equity, diversity and inclusion as well as limited economic resources, is imperative to understand how to integrate diabetes distress assessment across clinical services.

Interventions for adults with moderate-to-high diabetes distress often identify emotional regulation as a key pathway for reducing distress [76]. From an emotion-regulation framework, distress develops when the emotional demands of diabetes exceed a person’s capacity to identify, interpret, organise and adjust their diabetes-related emotions effectively. Burnout may therefore stem less from the demands of diabetes per se and more from how individuals respond to them [4, 77]. The if–then-because statements identified in this review consistently highlight emotional expression as a desirable outcome of care. Although comprehensive diabetes distress assessment alone may not significantly improve emotional regulation, it may serve as a catalyst by normalising emotional responses, prompting acknowledgement and disclosure and encouraging supported exploration. Assessment and discussion may therefore be necessary, although not sufficient, steps towards reducing diabetes distress by encouraging emotional disclosure enabled through person-centred questions and active listening. A future realist process evaluation will explore whether diabetes distress assessment and discussion improve emotional regulation skills and reduce the risk of escalation of diabetes distress.

### Strengths and limitations

An important strength of this review is the realist approach, which enabled the generation of causal explanations for how comprehensive assessment with discussion may work. Few included studies evaluated specific interventions or articulated causal pathways, so theories were generated through retroductive theorising and iteratively synthesising evidence and insights. These programme theories will be refined in a realist process evaluation of the D-Stress care pathway alongside a feasibility study and main trial, supported by ongoing stakeholder consultation. As per rapid realist review guidance, the search was not exhaustive; instead, expert input guided identification of key literature. Some relevant evidence may therefore have been missed. All included papers were from high-income countries, such as the UK, Australia and Denmark, limiting generalisability. Evidence about diabetes distress among minoritised and global populations is scarce, emphasising the need to test these theories in more diverse populations.

## Conclusions

This realist review provides detailed theories of how diabetes distress assessment and discussion may work in clinic consultations, although these require testing in practice. Variation is likely, given diversity among individuals and clinical settings. Understanding this complexity will strengthen our understanding of its effectiveness and how individual needs, experiences and contextual factors shape outcomes [78]. Future research should examine how contexts, mechanisms and outcomes interact in real-world settings and assess both the short- and long-term effectiveness and sustainability of comprehensive assessment of diabetes distress and discussion of emotional well-being in routine care.

## Supplementary Information

Below is the link to the electronic supplementary material.

Supplementary file1 (PDF 237 KB)

## Data Availability

All data and materials referenced in this manuscript are derived from previously published studies that are publicly available through the original sources.

## Abbreviations

CMOC: Context–mechanism–outcome configuration
HCP: Healthcare professional
PROM: Patient-reported outcome measure
RCT: Randomised controlled trial

## Code availability

All analyses were qualitative and based on previously published literature.

## References

[1] Fisher L, Polonsky WH, Hessler DM et al (2015) Understanding the sources of diabetes distress in adults with type 1 diabetes. J Diabetes Complications 29(4):572–577. 10.1016/j.jdiacomp.2015.01.012 PMC4414881 25765489

[2] Fisher L, Gonzalez JS, Polonsky WH (2014) The confusing tale of depression and distress in patients with diabetes: a call for greater clarity and precision. Diabet Med 31(7):764–772. 10.1111/dme.12428 PMC4065190 24606397

[3] Hessler DM, Fisher L, Polonsky WH et al (2017) Diabetes distress is linked with worsening diabetes management over time in adults with type 1 diabetes. Diabet Med 34(9):1228–1234. 10.1111/dme.13381 PMC5561505 28498610

[4] Fisher L, Hessler D, Polonsky W et al (2018) Emotion regulation contributes to the development of diabetes distress among adults with type 1 diabetes. Patient Educ Couns 101(1):124–131. 10.1016/j.pec.2017.06.036 PMC5732076 28739179

[5] Abdoli S, Hessler Jones D, Vora A, Stuckey H (2019) Improving diabetes care: should we reconceptualize diabetes burnout? Diabetes Educ 45:214–224. 10.1177/0145721719829066 30739546

[6] Fisher L, Hessler D, Polonsky W, Strycher L, Masharani U, Peters A (2016) Diabetes distress in adults with type 1 diabetes: prevalence, incidence and change over time. J Diabetes Complicat 30(6):1123–1128. 10.1016/j.jdiacomp.2016.03.032 PMC4949147 27118163

[7] Fisher L, Guzman S, Polonsky W, Hessler D (2024) Bringing the assessment and treatment of diabetes distress into the real world of clinical care: time for a shift in perspective. Diabet Med 41(12):e15446. 10.1111/dme.15446 39393003

[8] Hendrieckx C, Halliday JA, Russell-Green S et al (2020) Adults with diabetes distress often want to talk with their health professionals about it: findings from an audit of 4 Australian specialist diabetes clinics. Can J Diabetes 44(6):473–480. 10.1016/j.jcjd.2020.02.004 32360151

[9] Hex N, Bartlett C, Wright D, Taylor M, Varley D (2012) Estimating the current and future costs of type 1 and type 2 diabetes in the UK, including direct health costs and indirect societal and productivity costs. Diabet Med 29(7):855–862. 10.1111/j.1464-5491.2012.03698.x 22537247

[10] Stedman M, Lunt M, Davies M et al (2020) Cost of hospital treatment of type 1 diabetes (T1DM) and type 2 diabetes (T2DM) compared to the non-diabetes population: a detailed economic evaluation. BMJ Open 10(5):e033231. 10.1136/bmjopen-2019-033231 PMC7223153 32376746

[11] Roberts MZ, Scheiber FA, Moskovich AA, Merwin RM (2025) A scoping review of the relationship between psychological (in)flexibility and living with and managing type 1 and type 2 diabetes. Behav Sci (Basel) 15(6):792. 10.3390/bs15060792 PMC12189794 40564574

[12] Browne JL, Ventura A, Mosely K, Speight J (2013) ‘I call it the blame and shame disease’: a qualitative study about perceptions of social stigma surrounding type 2 diabetes. BMJ Open 3:e003384. 10.1136/bmjopen-2013-003384 PMC3840338 24247325

[13] Tanenbaum ML, Kane NS, Kenowitz J, Gonzalez JS (2016) Diabetes distress from the patient’s perspective: qualitative themes and treatment regimen differences among adults with type 2 diabetes. J Diabetes Complicat 30:1060–1068. 10.1016/j.jdiacomp.2016.04.023 PMC5792172 27217023

[14] Speight J, Hermanns N, Jensen et al (2026) EASD evidence-based clinical practice guideline for assessing and managing diabetes distress among adults with type 1 diabetes or type 2 diabetes. Diabetologia. 10.1007/s00125-026-06840-0 (in press)42744915

[15] Fisher L, Polonsky W, Naranjo D, Strycker L, Hessler D (2024) A novel approach to understanding and assessing the emotional side of type 1 diabetes: the Type 1-diabetes distress assessment system. Diabet Med 41:e15282. 10.1111/dme.15282 38244209

[16] Peck M, Papachristou Nadal I, Stenov V et al (2026) Introducing diabetes emotional wellbeing into routine consultations for adults with type 2 diabetes. Metabologia. 10.1007/s44357-026-00009-3 PMC13529831 42687914

[17] Saul JE, Willis CD, Bitz J, Best A (2013) A time-responsive tool for informing policy making: rapid realist review. Implement Sci 8:103. 10.1186/1748-5908-8-103 PMC3844485 24007206

[18] Wong G, Greenhalgh T, Westhorp G, Buckingham J, Pawson R (2013) RAMESES publication standards: realist syntheses. BMC Med 11(1):21. 10.1186/1741-7015-11-21 PMC3558331 23360677

[19] Greenhalgh T, Wong G, Westhorp G, Pawson R (2011) Protocol - realist and meta-narrative evidence synthesis: evolving standards (RAMESES). BMC Med Res Methodol 11(1):115. 10.1186/1471-2288-11-115 PMC3173389 21843376

[20] Wong G, Westhorp W, Pawson R, Greenhalgh T (2013) Realist synthesis. Rameses training materials. Available from: https://www.ramesesproject.org/media/Realist_reviews_training_materials.pdf. Accessed 19 Dec 2025

[21] Jagosh J, Macaulay AC, Pluye P et al (2012) Uncovering the benefits of participatory research: implications of a realist review for health research and practice. Milbank Q 90(2):311–346. 10.1111/j.1468-0009.2012.00665.x PMC3460206 22709390

[22] Astbury B, Leeuw FL (2010) Unpacking black boxes: mechanisms and theory building in evaluation. Am J Eval 31(3):363–381. 10.1177/1098214010371972

[23] Greenhalgh J, Manzano A (2022) Understanding ‘context’ in realist evaluation and synthesis. Int J Soc Res Methodol 25:583–595. 10.1080/13645579.2021.1918484

[24] Greenhalgh T, Pawson R, Wong G, Jagosh J (2017) Retroduction in realist evaluation. The Rameses II project. Available from: https://www.ramesesproject.org/media/RAMESES_II_Retroduction.pdf. Accessed 19 Dec 2025

[25] Pawson R (2006) Digging for nuggets: how ‘bad’ research can yield ‘good’ evidence. Int J Soc Res Methodol 9(2):127–142. 10.1080/13645570600595314

[26] Pawson R, Greenhalgh T, Harvey G, Walshe K (2004) Realist synthesis: an introduction. ESRC research methods programme, methods paper 2. Available from: https://www.betterevaluation.org/sites/default/files/RMPmethods2.pdf

[27] Pawson R, Tilley N (1997) Realistic evaluation. SAGE, London

[28] Schipper SBJ, Karagiannis T, Zaccardi F (2026) Interventions to reduce diabetes-related distress among adults with type 1 or type 2 diabetes: a systematic review and meta-analysis of randomised controlled trials. Diabetologia. 10.1007/s00125-026-06789-042726276

[29] Hewitt G, Sims S, Harris R (2012) The realist approach to evaluation research: an introduction. Int J Ther Rehabil 19:250–260. 10.12968/ijtr.2012.19.5.250

[30] Benton M, Baykoca J, Ismail K, Price H (2023) Healthcare professionals’ experiences in identifying and supporting mental health problems in adults living with type 1 diabetes mellitus: a qualitative study. Diabet Med 40(7):e15103. 10.1111/dme.15103 37004151

[31] Health Innovation Network (n.d.) Diabetes distress screening scale (DDS17). Available from: https://healthinnovationnetwork.com/wp-content/uploads/2017/01/Diabetes-Ditress-Screening-Scale.pdf. Accessed 19 Dec 2025

[32] Due-Christensen M, Zoffman V, Hommel E, Lau M (2012) Can sharing experiences in groups reduce the burden of living with diabetes, regardless of glycaemic control? Diabet Med 29(2):251–256. 10.1111/j.1464-5491.2011.03521.x 22061095

[33] Due-Christensen M, Zoffman V, Willaing W, Hopkins D, Forbes A (2018) The process of adaptation following a new diagnosis of type 1 diabetes in adulthood: a meta-synthesis. Qual Health Res 28(2):245–258. 10.1177/1049732317745100 29235942

[34] Due-Christensen M, Joensen LE, Sarre S et al (2021) A co-design study to develop supportive interventions to improve psychological and social adaptation among adults with new-onset type 1 diabetes in Denmark and the UK. BMJ Open 11(11):e051430. 10.1136/bmjopen-2021-051430 PMC8565545 34728449

[35] Due-Christensen M, El Hakim R, Piper D et al (2026) Psychosocial support for adult-onset type 1 diabetes: the living with and adapting to type 1 diabetes programme - a cross-national feasibility study. Front Clin Diabetes Healthc 7:1749766. 10.3389/fcdhc.2026.1749766 PMC13035499 41924325

[36] Due-Christensen, M (2024) Sustain – prevention of diabetes distress. The living with and adapting to diabetes programme. Psychosocial support for adults with new-onset type 1 diabetes in public health care systems in Denmark and the UK 2024. D-Stress Study Kick-off conference, 1–2 May 2024, Burnham, UK. Available from: https://www.kcl.ac.uk/nmpc/assets/research/projects/due-christensen-sustain-presentation-2024.pdf. Accessed 1 Jul 2026

[37] Pals RAS, Cleal B, Forbes A, Due-Christensen M (2026) Developing initial programme theories for a realist evaluation of an intervention to support adults in adapting to life with type 1 diabetes: methodological insights and implications for practice. Int J Qual Methods. 10.1177/16094069261431989

[38] Halliday JA, Speight J, Bennett A, Beeney LJ, Hendrieckx C (2020) The diabetes and emotional health handbook and toolkit for health professionals supporting adults with type 1 and type 2 diabetes: formative evaluation. JMIR Form Res 4(2):e15007. 10.2196/15007 PMC7060499 32130112

[39] Halliday JA, Speight J, Russell-Green S et al (2021) Developing a novel diabetes distress e-learning program for diabetes educators: an intervention mapping approach. Transl Behav Med 11(6):1264–1273. 10.1093/tbm/ibaa144 33677509

[40] Halliday, J. A (2024) An eLearning to upskill health professionals in providing support for diabetes distress. D-Stress Study Kick-off conference, 1–2 May 2024, Burnham, UK. Available from: https://www.kcl.ac.uk/nmpc/assets/research/projects/halliday-presentation-2024.pdf. Accessed 1 Jul 2026

[41] Health Innovation Network (n.d.) Type 1 diabetes consultation tool. Available from: https://healthinnovationnetwork.com/wp-content/uploads/2017/01/Type-1-Consultation-Tool-Large-Print.pdf. Accessed 19 Dec 2025

[42] Hernar I, Graue M, Richards DA et al (2021) Use of patient-reported outcome measures (PROMs) in clinical diabetes consultations: the DiaPROM randomised controlled pilot trial. BMJ Open 11(4):e042353. 10.1136/bmjopen-2020-042353 PMC8054082 33853796

[43] Holt RIG, DeVries JH, Hess-Fischl A et al (2021) The management of type 1 diabetes in adults. A consensus report by the American Diabetes Association (ADA) and the European Association for the Study of Diabetes (EASD). Diabetologia 64(12):2609–2652. 10.1007/s00125-021-05568-3 PMC8481000 34590174

[44] Litterbach E, Holmes-Truscott E, Pouwer F, Speight J, Hendrieckx C (2020) “I wish my health professionals understood that it’s not just all about your HbA(1c)!”. Qualitative responses from the second Diabetes MILES - Australia (MILES-2) study. Diabet Med 37(6):971–981. 10.1111/dme.14199 31802530

[45] Lowry M, Morrissey EC, Dinneen SF (2021) Piloting an intervention to improve outcomes in young adults living with type 1 diabetes: the experience of the D1 now support worker. Front Clin Diabetes Healthc 2:799589. 10.3389/fcdhc.2021.799589 PMC10012156 36994338

[46] Luong D, Griffin A, Barrett BL, Hendrieckx C, D’Silva N (2023) Emotional well-being and HbA1c following the implementation of the Diabetes Psychosocial Assessment Tool (DPAT) in young adults with type 1 diabetes (T1DM): An observational study. Diabetes Res Clin Pract 200:110696. 10.1016/j.diabres.2023.110696 37164160

[47] Mosely K, Aslam A, Speight J (2010) Overcoming barriers to diabetes care: perceived communication issues of healthcare professionals attending a pilot Diabetes UK training programme. Diabetes Res Clin Pract 87(2):e11–e14. 10.1016/j.diabres.2009.12.003 20044163

[48] Nygaard M, Willaing I, Joensen LE et al (2023) A short-form measure of diabetes distress among adults with type 1 diabetes for use in clinical practice: development and validation of the T1-DDS-7. Diabetes Care 46(9):1619–1625. 10.2337/dc23-0460 37343387

[49] Orben K, Ritholz MD, McCalla M, Beverly EA (2022) Differences and similarities in the experience of living with diabetes distress: a qualitative study of adults with type 1 and type 2 diabetes. Diabet Med 39(10):e14919. 10.1111/dme.14919 35842933

[50] Problem Area in Diabetes-Scale (PAID). Available from: https://professional.diabetes.org/sites/default/files/media/ada_mental_health_toolkit_questionnaires.pdf. Accessed 19 Dec 2025

[51] Schultz AA, Wad JL, Willaing I, Nørgaard K, Persson F, Joensen LE (2021) Achieving a useful and person-centred diabetes consultation is a shared responsibility between diabetologists and people with diabetes: a qualitative study of perspectives from people with type 1 diabetes. Diabet Med 38(6):e14382. 10.1111/dme.14382 33245572

[52] Sturt J, Choudhary P (2026) The impact of a holistic consultation framework for type 1 diabetes (T1C) on diabetes distress, HbA1c and hypoglycaemia awareness in routine clinical care. Working paper/Preprint, King’s College London, London, UK. Available from: https://kclpure.kcl.ac.uk/portal/en/publications/the-impact-of-a-holistic-consultation-framework-for-type-1-diabet/. Accessed 1 Jul 2026

[53] Skinner TC, Joensen L, Parkin T (2020) Twenty-five years of diabetes distress research. Diabet Med 37(3):393–400. 10.1111/dme.14157 31638279

[54] Stenov V, Due-Christensen M, Christensen JN, Willaing I, Cleal B (2025) Discovering the hidden emotional burden: systematic screening for diabetes distress in adults with type 1 diabetes in nurse-led routine diabetes care. Diabet Med. 10.1111/dme.70064 PMC12151820 40373169

[55] Todd PJ, Edwards F, Spratling L et al (2018) Evaluating the relationships of hypoglycaemia and HbA1c with screening-detected diabetes distress in type 1 diabetes. Endocrinol Diabetes Metab 1(1):e00003. 10.1002/edm2.3 PMC6353214 30815540

[56] Type 1 Diabetes Distress Scale (T1-DDS). Available from: https://diabetesdistress.org/dd-assess-score-2/. Accessed 19 Dec 2025

[57] Type 1 Diabetes Distress Assessment System (T1-DDAS). Available from: https://diabetesdistress.org/dd-assess-score-5/. Accessed 19 Dec 2025 10.1111/dme.70066 40341664

[58] Yared Z, Blunden S, Stotland S (2020) Addressing a care gap in type 1 diabetes management: using the Diabetes Distress Scale in a community care setting to address diabetes-related treatment challenges. Can J Diabetes 44(6):514–520. 10.1016/j.jcjd.2020.06.011 32792105

[59] Zoffmann V, Vistisen VD, Due-Christensen M (2015) Flexible guided self-determination intervention for younger adults with poorly controlled Type 1 diabetes, decreased HbA1c and psychosocial distress in women but not in men: a real-life RCT. Diabet Med 32(9):1239–1246. 10.1111/dme.12698 25601214

[60] Hendrieckx C, Halliday JA, Beeney LJ, Speight J (2019) Diabetes and emotional health: a practical guide for healthcare professionals supporting adults with type 1 and type 2 diabetes, 2nd edn. Deakin University, London https://hdl.handle.net/10536/DRO/DU:30120077 10.2196/15007 PMC7060499 32130112

[61] Chew BH, Vos RC, Stellato RK, Ismail M, Rutten GEHM (2018) The effectiveness of an emotion-focused educational programme in reducing diabetes distress in adults with Type 2 diabetes mellitus (VEMOFIT): a cluster randomized controlled trial. Diabet Med 35(6):750–759. 10.1111/dme.13615 29505098

[62] Chew BH, Vos RC, Fernandez A et al (2019) The effectiveness of an emotion-focused educational programme in reducing diabetes distress in adults with type 2 diabetes mellitus at 12-month follow-up: a cluster randomized controlled trial. Ther Adv Endocrinol Metab 10:2042018819853761. 10.1177/2042018819853761 PMC6545652 31210922

[63] Clark D, Sundeen E, Jenkins C (2024) Diabetes self-management support through diabetes-related distress awareness. J Nurse Pract 20(8):105129. 10.1016/j.nurpra.2024.105129

[64] Dennick K, Sturt J, Speight J (2017) What is diabetes distress and how can we measure it? A narrative review and conceptual model. J Diabetes Complicat 31(5):898–911. 10.1016/j.jdiacomp.2016.12.018 28274681

[65] Due-Christensen M, Hommel E, Ridderstråle M (2016) Potential positive impact of group-based diabetes dialogue meetings on diabetes distress and glucose control in people with type 1 diabetes. Patient Educ Couns 99(12):1978–1983. 10.1016/j.pec.2016.07.023 27444233

[66] Due-Christensen M, Willaing I, Ismail K, Forbes A (2019) Learning about type 1 diabetes and learning to live with it when diagnosed in adulthood: two distinct but inter-related psychological processes of adaptation A qualitative longitudinal study. Diabet Med 36(6):742–752. 10.1111/dme.13838 30329176

[67] Embaye J, de Wit M, Snoek F (2024) A self-guided web-based app (MyDiaMate) for enhancing mental health in adults with type 1 diabetes: insights from a real-world study in the Netherlands. JMIR Diabetes 9:e52923. 10.2196/52923 PMC11024740 38568733

[68] Halliday JA, Russell-Green S, Hagger V et al (2022) Feasibility and acceptability of e-learning to upskill diabetes educators in supporting people experiencing diabetes distress: a pilot randomised controlled trial. BMC Med Educ 22(1):768. 10.1186/s12909-022-03821-w PMC9644574 36352377

[69] Muijs LT, de Wit M, Knoop H, Snoek FJ (2021) Feasibility and user experience of the unguided web-based self-help app “MyDiaMate” aimed to prevent and reduce psychological distress and fatigue in adults with diabetes. Internet Interv 25:100414. 10.1016/j.invent.2021.100414 PMC8350600 34401373

[70] Polonsky WH, Anderson BJ, Lohrer PA et al (1995) Assessment of diabetes-related distress. Diabetes Care 18(6):754–760. 10.2337/diacare.18.6.754 7555499

[71] Shah S, Todd P, Chaudary P (2017) Improvements in diabetes distress are associated with improvements in biomedical outcomes in type 1 diabetes. Diabetologia 66(Suppl 1):S64. 10.1007/s00125-017-4350-z

[72] Schmidt CB, Potter van Loon BJ, Vergouwen ACM, Snoek FJ, Honig A (2018) Systematic review and meta-analysis of psychological interventions in people with diabetes and elevated diabetes-distress. Diabet Med 35(9):1157–1172. 10.1111/dme.13709 29896760

[73] Sturt J, Dennick K, Due-Christensen M, McCarthy K (2015) The detection and management of diabetes distress in people with type 1 diabetes. Curr Diab Rep 15(11):101. 10.1007/s11892-015-0660-z 26411924

[74] Kors JM, Paternotte E, Martin L et al (2020) Factors influencing autonomy supportive consultation: a realist review. Patient Educ Couns 103(10):2069–2077. 10.1016/j.pec.2020.04.019 32471798

[75] Jagosh J, Bush PL, Salsberg J et al (2015) A realist evaluation of community-based participatory research: partnership synergy, trust building and related ripple effects. BMC Public Health 15:725. 10.1186/s12889-015-1949-1 PMC4520009 26223523

[76] Fisher L, Hessler D, Polonsky W, Strycker L, Bowyer V, Masharani U (2019) Toward effective interventions to reduce diabetes distress among adults with type 1 diabetes: enhancing emotion regulation and cognitive skills. Patient Educ Couns 102(8):1499–1505. 10.1016/j.pec.2019.03.021 PMC6565487 30952482

[77] Coccaro EF, Lazarus S, Joseph J et al (2021) Emotional regulation and diabetes distress in adults with type 1 and type 2 diabetes. Diabetes Care 44(1):20–25. 10.2337/dc20-1059 PMC8742145 33444157

[78] Skivington K, Matthews L, Simpson SA et al (2021) A new framework for developing and evaluating complex interventions: update of Medical Research Council guidance. BMJ 374:n2061. 10.1136/bmj.n2061 PMC8482308 34593508

