# Supplementary material for: Introducing diabetes emotional well-being into routine consultations for adults with type 1 diabetes
Source: Metabologia. 2026 Aug 31;2(1):10. doi: 10.1007/s44357-026-00010-w (PMC13529843; doi:10.1007/s44357-026-00010-w)
Supplement: Supplementary file 1 [file 44357_2026_10_MOESM1_ESM.pdf]

Title: Introducing diabetes emotional wellbeing into routine consultations for adults with type 1 diabetes

Journal name: Metabologia

Authors: Ruth Harris, Sarah Sims, Mette Due-Christensen, Vibeke Stenov, Ruth Abrams, Clara Fabian-Therond, Shalini Ahuja, Siobhan O'Connor, Iliatha Papachristou Nadal, Megan Peck, Jane Speight, Richard I. G. Holt, Jackie Sturt, and the D-Stress study group

Corresponding Author: Ruth Harris, King's College London, ruth.harris@kcl.ac.uk

**ESM Box 1. Search strategy for searches in MEDLINE, Embase, HMIC and PsycINFO**
**Free text terms and operators**<br>*"acceptance and commitment therapy"* OR *"bio psycho social model"* OR *"motivational interviewing"* OR *"self determination theory"* OR *"empowerment based communication"* OR *"peer support"* OR *"social learning theory"* OR *"social comparison theory"*<br>AND<br>*"realist"*<br>[**Limiters:** English language only]

ESM Table 1 - Included literature (n=35).

Author and date	Title of paper	Country of publication	Paper type
Benton et al. 2023 [30]	Healthcare professionals' experiences in identifying and supporting mental health problems in adults living with type 1 diabetes mellitus: a qualitative study	UK	Research paper
Due-Christensen et al. 2012 [32]	Can sharing experiences in groups reduce the burden of living with diabetes, regardless of glycaemic control?	Denmark	Research paper
Due-Christensen et al. 2018 [33]	The Process of Adaptation Following a New Diagnosis of Type 1 Diabetes in Adulthood: A Meta-Synthesis.	UK	Research paper
Due-Christensen et al. 2021 [34]	A co-design study to develop supportive interventions to improve psychological and social adaption mong adults with new-onset type 1 diabetes in Denmark and the UK.	Denmark	Research paper
Due-Christensen et al 2026 [35]	Psychosocial support for adult-onset type 1 diabetes: The Living with and adapting to Type 1 Diabetes Programme – a cross-national feasibility study.	Denmark	Research paper
Due-Christensen et al. 2024 [36]	SUSTAIN – prevention of diabetes distress	Denmark	Powerpoint presentation

Fisher et al. 2015 [1]	Understanding the sources of diabetes distress in adults with type 1 diabetes.	US	Research paper
Fisher et al. 2018 [4]	Emotion regulation contributes to the development of diabetes distress among adults with type diabetes	US	Research paper
Fisher et al 2024 [7]	Bringing the assessment and treatment of diabetes distress into the real world of clinical care: time for a shift in perspective.	US	Research paper
Halliday et al. 2020 [38]	The Diabetes and Emotional Health handbook and toolkit for health professionals supporting adults with type1 and type 2 diabetes: formative evaluation.	Australia	Research paper
Halliday et al. 2021 [39]	Developing a novel diabetes distress e-learning program for diabetes educators: an intervention mapping approach.	Australia	Research paper
Halliday et al. 2024 [40]	An eLearning to upskill health professionals in providing support for diabetes distress	Australia	Powerpoint presentation
Hendrieckx et al. 2019 [60]	Diabetes and emotional health: a practical guide for health professionals supporting adults with Type 1 and Type 2 diabetes.	UK	Practical guide
Hernar et al. 2021 [42]	Use of patient-reported outcome measures (PROMs) in clinical diabetes consultations: the DiaPROM randomised controlled pilot trial.	Norway	Research paper
Holt et al. 2021 [43]	The management of type 1 diabetes in adults. A consensus report by the American Diabetes Associate (ADA) and the European Association for the Study of Diabetes (EASD).	UK	Consensus statement
Litterbach et al. 2020 [44]	‘I wish my health professionals understood that it’s not just about your HbA1c!’ Qualitative responses from the second Diabetes MILES – Australis (MILES-2) study.	Australia	Research paper
Lowry et al. 2021 [45]	Piloting an intervention to improve outcomes in young adults living with type 1 diabetes: the experience of the D1 Now Support Worker.	Ireland	Research paper
Luong et al. 2023 [46]	Emotional well-being and HbA1c following the implementation of the Diabetes Psychosocial Assessment Tool (DPAT) in young adults with type 1 diabetes (T1DM): an observational study.	Australia	Research paper
Mosely et al. 2010 [47]	Overcoming barriers to diabetes care: Perceived communication issues of healthcare professionals attending a pilot Diabetes UK training programme.	UK	Research paper
Nygaard et al. 2023 [48]	A short-form measure of diabetes distress among adults with type 1 diabetes for use in clinical practice: development and validation of the T1-DDS-7	Denmark	Research paper
Orben et al. 2022 [49]	Differences and similarities in the experience of living with diabetes distress: a qualitative study of adults with type 1 and type 2 diabetes.	US	Research paper

Pals et al. 2026 [37]	Developing initial programme theories for a realist evaluation of an intervention to support adults in adapting to life with type 1 diabetes: methodological insights and implication for practice.	Denmark	Research paper
Schultz et al. 2021 [51]	Achieving a useful and person-centred diabetes consultation is a shared responsibility between diabetologists and people with diabetes: a qualitative study of perspectives from people with type 1 diabetes.	Denmark	Research paper
Sturt and Choudhary (unpublished) [52]	The impact of a holistic consultation framework for type 1 diabetes (T1D) on diabetes distress, HbA1c and hypoglycaemia awareness in routine clinical care.	UK	Research working paper (unpublished)
Skinner et al. 2020 [53]	Twenty-five years of diabetes distress research	Denmark	Review paper
Stenov et al. 2025 [54]	Discovering the hidden emotional burden: systematic screening for diabetes distress in adults with type 1 diabetes in nurse-led routine diabetes care.	Denmark	Research paper
Todd et al. 2018 [55]	Evaluating the relationships of hypoglycaemia and HbA1c with screening-detected diabetes distress in type 1 diabetes.	UK	Research paper
Yared et al. 2020 [58]	Addressing a care gap in type 1 diabetes management: using the Diabetes Distress Scale in a community setting to address diabetes-related treatment challenges.	Canada	Research paper
Health Innovation Network 2017 [31]	Health Innovation Network. Diabetes Distress Screening Scale (DDS17)	UK	Assessment Scale
King's Health Partners & Health Innovation Network South London (no date) [41]	Type 1 Diabetes Consultation Tool	UK	Consultation tool
American Diabetes Association, 2021 [50]	Problem Areas In Diabetes (PAID) Scale	US	Assessment Scale
Behavioral Diabetes Institute (2016) [56]	Type 1 Diabetes Distress Scale (T1-DDS)	US	Assessment Scale
Behavioral Diabetes Institute (2023) [57]	Type 1 Diabetes Distress Assessment System (T1DDAS)	US	Assessment Scale
Zoffman et al. (2015) [59]	Flexible guided self-determination intervention for younger adults with poorly controlled Type 1 diabetes, decreased HbA1C and psychosocial distress in women but not in men: a real-life RCT.	Denmark	Research paper

Kors et al. 2020 [74]	Factors influencing autonomy supportive consultation: a realist review.	Netherlands	Review paper
